# On the Pivotal Role of Veterinarians in the Fight Against Avian Influenza A (H5N1) Virus, COVID-19 and Future Pandemics

**DOI:** 10.3390/pathogens14040340

**Published:** 2025-03-31

**Authors:** Giovanni Di Guardo

**Affiliations:** General Pathology and Veterinary Pathophysiology, Faculty of Veterinary Medicine, University of Teramo, 64100 Teramo, Italy; gdiguardo@unite.it

An interesting article recently published in *Science* highlights the actions, the strategies and the tools through which the potential emergence of a new pandemic caused by the avian influenza A (H5N1) virus could be efficiently counteracted. This approach would also apply to other pandemic threats emerging in the more or less near future on the global scene, thereby gaining benefit from the lessons learned during the COVID-19 pandemic, with special emphasis on the revolutionary mRNA technology-based vaccines, which have saved millions of lives [1].

Notwithstanding the above, however, we should firmly keep in mind that both the avian influenza A (H5N1) virus and the SARS-CoV-2 betacoronavirus—the agent responsible for COVID-19—are zoonotic pathogens, similar to approximately 70% of “emerging infectious diseases”, whose proven or suspect origin would lie in one or more animal reservoirs [2].

This allows me to underscore the everlasting concepts/principles of “*One Health, One Earth, One Ocean*”, as the COVID-19 pandemic has clearly taught us, thus making absolutely imperative an intersectorial and multidisciplinary collaboration effort primarily (but not exclusively) involving physicians and veterinarians.

As a veterinarian with a long teaching and research experience in comparative pathology, I would also like to recall herein the “historical reason” anticipating the birth of the most ancient European veterinary medical schools, such as those of Lyon, Turin, and Bologna, founded in 1761, 1769, and 1784 in France and Italy, respectively. Such a historical reason was represented, in fact, by “rinderpest”, a highly contagious morbilliviral disease, now eradicated globally, by which cattle herds were heavily affected during the 18th century all over the so-called “Old Continent”. This has unavoidably implied, and still continues to imply, that the study of animal infectious diseases, of both zoonotic and non-zoonotic nature, is of paramount relevance for veterinary students and professionals, with a strong “disease prevention-based mentality” generally characterizing our careers and activities.

Within this framework, since the financial resources needed for prevention are exceedingly lower compared to those allowing for disease therapy, the aforementioned educational and professional background of veterinarians undoubtedly represents a powerful weapon to efficiently counteract and even foresee, in some instances, the appearance and the subsequent spread of new, emerging, and potentially pandemic pathogens from animals to mankind and vice versa, as well as between human beings.

Regretfully enough, however, the significant contribution “historically” brought by veterinarians to the fight against zoonotic infectious diseases often appears to be overlooked, especially by the general public, with a lot of people having a limited knowledge of, if not largely ignoring, “what” veterinarians truly “do” in their professional life.

Furthermore, no veterinarians were included in the “COVID-19 Scientific Committee”, which was dismantled two years after it was officially set up by the Italian Government in order to tackle and monitor SARS-CoV-2 infection’s spread and evolution [3].

“*Historia magistra vitae*” and, no less important, “*Errare humanum est perseverare autem diabolicum*”!
